# Macrophage activation syndrome in MDA5 antibody-positive dermatomyositis and COVID-19 infection

**DOI:** 10.1186/s41927-021-00225-z

**Published:** 2021-12-13

**Authors:** Marzieh Keshtkarjahromi, Sumit Chhetri, Amulya Balagani, Umm-ul-Banin B. Tayyab, Christopher J. Haas

**Affiliations:** 1 MedStar Health Internal Medicine Residency Program, Baltimore, MD USA; 2 Georgetown University School of Medicine, Washington, DC USA

**Keywords:** Dermatomyositis, Macrophage activation syndrome, COVID-19 infection

## Abstract

**Background:**

Macrophage activation syndrome (MAS) is a rare multiorgan system disorder that may present as a fatal complication of underlying rheumatological disease, including dermatomyositis.

**Case presentation:**

Here, we report the case of a 65-year-old Caucasian female with a history of psoriasis and a recent diagnosis of Coronavirus disease 2019 (COVID-19) who presented with progressive generalized weakness, joint pains, an erythematous rash, shortness of breath, and weight loss. She was ultimately diagnosed with biopsy-confirmed melanoma differentiation-associated protein 5 (MDA5)-positive dermatomyositis complicated by MAS, requiring intravenous immunoglobulin and high-dose methylprednisolone.

**Conclusions:**

This report serves as a clinical reminder of the rare, yet clinically relevant association between MDA5-positive dermatomyositis and MAS, as well as highlights the potential contribution of other immune system activating diseases, such as COVID-19, associated with a cytokine storm and hyperinflammatory state.

## Background

Macrophage activation syndrome (MAS)/secondary hemophagocytic lymphohistiocytosis (HLH) is a potentially life-threatening complication of systemic inflammatory disorders characterized by excessive activation and expansion of macrophages and T lymphocytes [1]. It is commonly associated with systemic juvenile idiopathic arthritis (SJIA), adult onset still disease, Systemic lupus erythematous (SLE), Kawasaki disease, and rarely, dermatomyositis [1–3]. Prior reports have demonstrated the presence of activated macrophages and hemophagocytic histiocytes in patients with rheumatic disease, labeling the disorder reactive hemophagocytic syndrome, now known as MAS [4].

HLH is divided in two categories: primary/ familial and secondary/ acquired. Primary HLH is an inherited disease associated with inherited immunodeficiencies, whereas secondary is triggered by an underlying disease such as infection (most commonly viral), autoimmune, or neoplastic processes. MAS considered a subtype of secondary HLH that is specifically associated with underlying rheumatological disorders and is characterized by chronic immune dysregulation [1, 5]. The pathophysiology of MAS is hypothesized to be secondary to an uninterrupted hyperstimulation of the immune system with resultant activation of T lymphocytes and hemophagocytic macrophages. Laboratory manifestation include cytopenias, liver dysfunction, coagulopathy and hyperferritinemia [1]. Clinical manifestations include prolonged fever, hepatomegaly, splenomegaly, lymphadenopathy, and hemorrhagic features (purpura, easy bruising, or mucosal bleeding). While multiple diagnostic criteria have been developed for HLH—HLH-2004 criteria (Table 1), the preliminary 2016 classification criteria for SJIA complicated by MAS, and MAS as a complication of SLE – there is no universally accepted criteria, with the HLH-2004 criteria most often used. The diagnosis of MAS is often delayed given the non-specific clinical and laboratory diagnostic findings and similarity to other disease processes, leading to increased morbidity and mortality. Early diagnosis and initiation of therapy is key to prevent morbidity and mortality [2, 6–8].

**Table 1:** Diagnostic criteria for HLH used in HLH-2004 trial [9]

A	Molecular diagnosis consistent with HLH associated mutations (PRF1, UNC13D, STX11, STXBP2, Rab27A, SHD1A, BIRC4, LYST, ITK, SLC7A7, XMEN, HPS)
B	Or 5 of 8 criteria listed below
1	Fever ≥ 38.5 °C
2	Splenomegaly
3	Peripheral blood cytopenia, with at least two of the following: hemoglobin < 9 g/dL (for infants < 4 weeks, hemoglobin < 10 g/dL); platelets < 100,000/microL; absolute neutrophil count < 1000/microL
4	Hypertriglyceridemia (fasting triglycerides > 265 mg/dL) and/or hypofibrinogenemia (fibrinogen < 150 mg/dL)
5	Hemophagocytosis in bone marrow, spleen, lymph node, or liver
6	Low or absent NK cell activity
7	Ferritin > 500 ng/mL
8	Elevated soluble CD25 (soluble IL-2 receptor alpha [sIL-2R]) two standard deviations

## Case presentation

A 65-year-old Caucasian female with history of psoriasis, hypertension, hyperlipidemia, and recent diagnosis of COVID-19 infection two months prior, presented with progressive generalized weakness, weight loss, skin rash, and shortness of breath of three months duration. She described a progressive intolerance not only to climbing stairs, but also rising from a seated position. Associated symptoms included arthralgia of the bilateral wrists and new rashes of the bilateral hands, chest, back, and around the eyes. She experienced progressively worsening shortness of breath, without evidence of lower extremity edema, orthopnea, or paroxysmal nocturnal dyspnea. Her home medications included Amlodipine, Atorvastatin, Buspirone, and Pantoprazole. There were no recent changes in her home medications. No recent travel, sick contacts, or additional exposures were reported. Family history was unremarkable.

On presentation, the patient was tachycardic (118 beats per min), tachypneic (30 breaths per minute), normotensive (112/60 mmHg), and saturating 93–98% on room air. Clinical examination was remarkable for a heliotrope rash, erythematous rash on of the upper back (Shawl sign) and chest (V sign), Gottron’s papules on the hands and elbows, periungual erythema, and right wrist swelling. She exhibited symmetric bilateral deltoid and iliopsoas weakness. Laboratory diagnostics revealed leukopenia, an elevated aspartate aminotransferase (AST), alanine aminotransferase (ALT), alkaline phosphatase (ALP), and a normal bilirubin. Creatine kinase (CK), lactate dehydrogenase (LDH), C-reactive protein (CRP) were elevated with a notably normal erythrocyte sedimentation rate (ESR) (Table 2).

**Table 2:** Laboratory findings

	Laboratory findings on first presentation	Laboratory findings at discharge	Laboratory findings on second presentation	Reference Range
WBC	3.7 k/µL	4.3 k/µL	2.0 k/µL	4–10.8 k/µL
Absolute lymphocyte count	0.6 k/µL	0.2 k/µL	0.1 k/µL	0.6–4.9 k/µL
Hemoglobin	14.2 gm/dL	10.2 gm/dL	10.1 gm/dL	11–14.5 gm/dL
Blood urea nitrogen	20 mg/dL	22 mg/dL	50 mg/dL	7–17 mg/dL
Creatinine	1.30 mg/dL	0.97 mg/dL	2.34 mg/dL	0.52–1.04 mg/dL
Aspartate aminotransferase (AST)	179 units/L	176 units/L	497 untis/L	3–35 units/L
Alanine aminotransferase (ALT)	55 units/L	171 units/L	121 units/L	15–41 units/L
Alkaline phosphatase (ALP)	180 units/L	267 units/L	279 units/L	40–117 units/L
Total Bilirubin	0.8 mg/dl	0.6 mg/dl	0.9 mg/dl	0.2–1.3 mg/dl
Creatine Kinase	1222 units/L	267 units/L	441 units/L	26–192 units/L
Lactate Dehydrogenase	450 units/L		1727 units/L	86–246 units/L
C-reactive protein	17 mg/L		67 mg/L	0–10 mg/L
Erythrocyte sedimentation rate	11 mm/h		20 mm/h	0–30 mm/h
ANA screen	Positive, speckled pattern			
ANA titer	1:80			
MDA5(CADM-140)	High Positive			
C3	78 mg/dl			
SSA 52 (Ro) (ENA)IgG	107 AU/ml			
Ferritin			> 16,500 ng/mL	5–148 ng/mL
Triglyceride			461 mg/dl	0–149 mg/dl
Interleukin 2 receptor (CD-25)			365.3 pg/mL	175.3–858.2 pg/mL
Fibrinogen			259 mg/dL	213–536 mg/dL

**Fig. 1:**
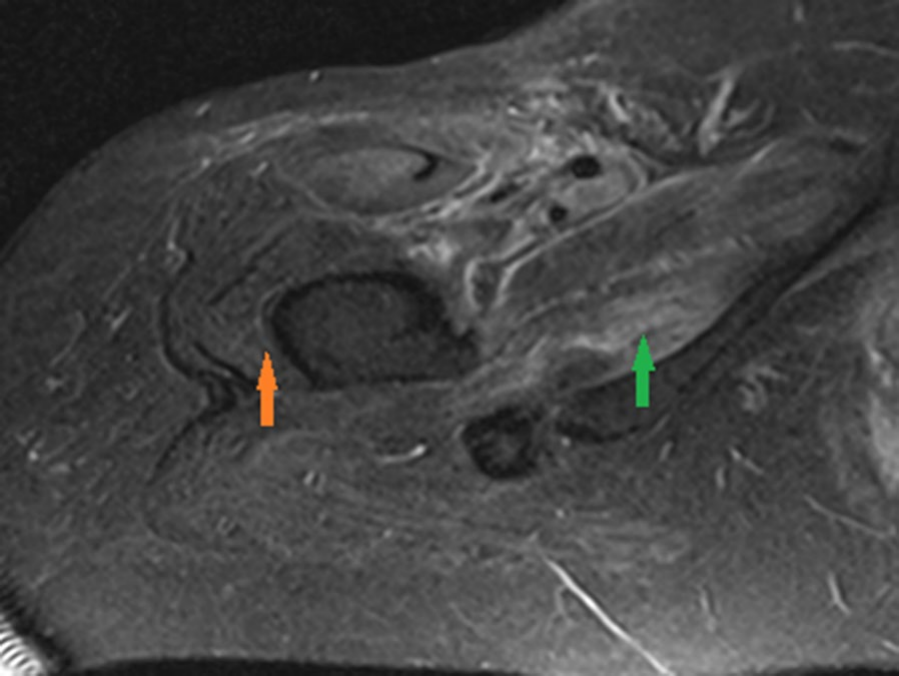
Magnetic resonance imaging (MRI) of the right femur in axial inversion recovery sequence image demonstrates extensive signal abnormality within the right hip muscles which shows features of muscle edema (green arrow). The orange arrow demonstrates the normal muscle

**Fig. 2:**
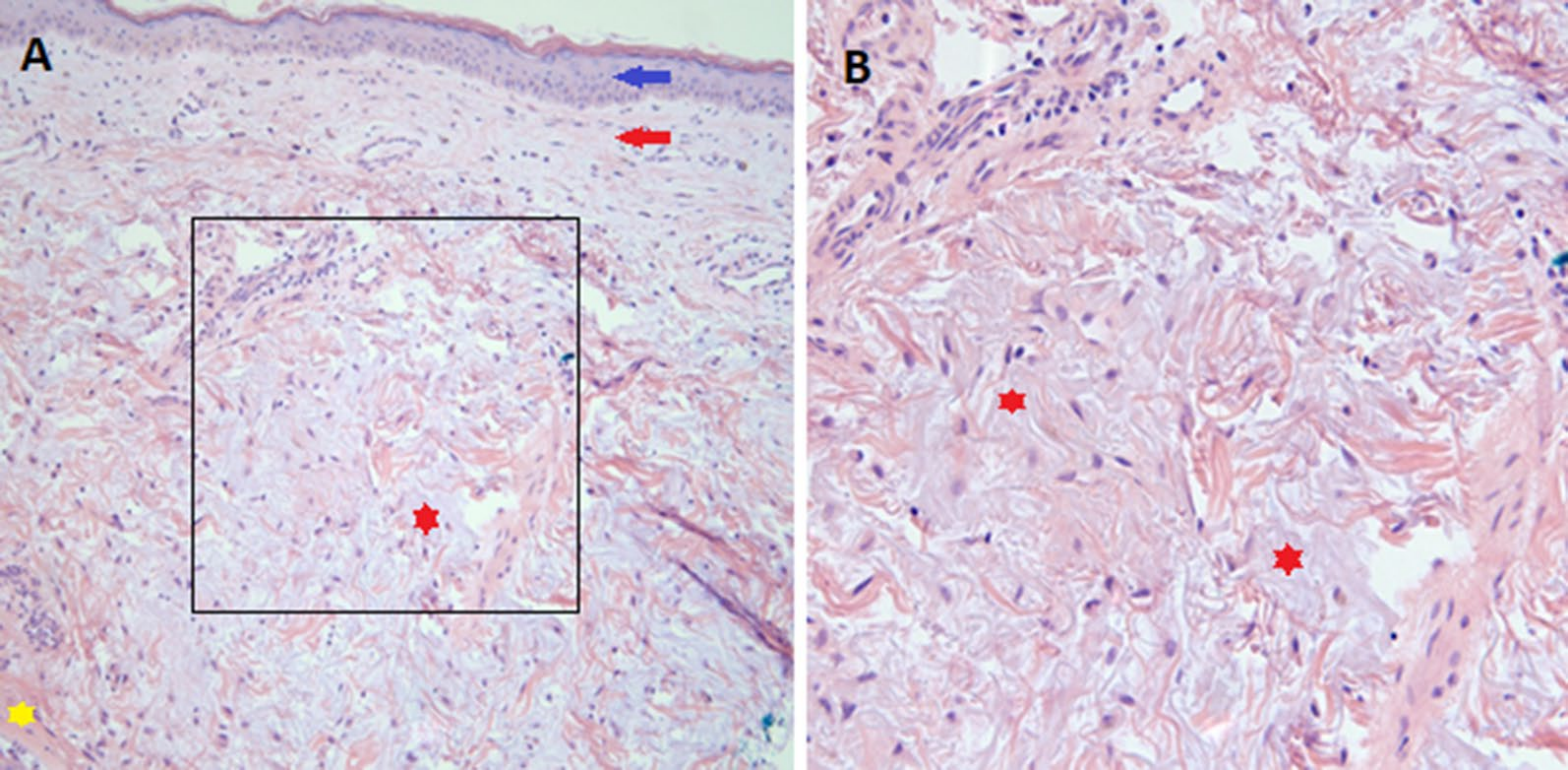
Mild vacuolar interface dermatitis within an increase in dermal mucin. Hematoxylin and eosin stain of the skin biopsy. Blue and red arrows in **a** demonstrate epidermis and dermis, respectively. Yellow star demonstrates the normal pinkish dermis and the red star demonstrates the grayish-purplish mucin deposition. **b** Demonstrates a magnified view of the black box in **a** which demonstrates the dermal mucin deposition

**Fig. 3:**
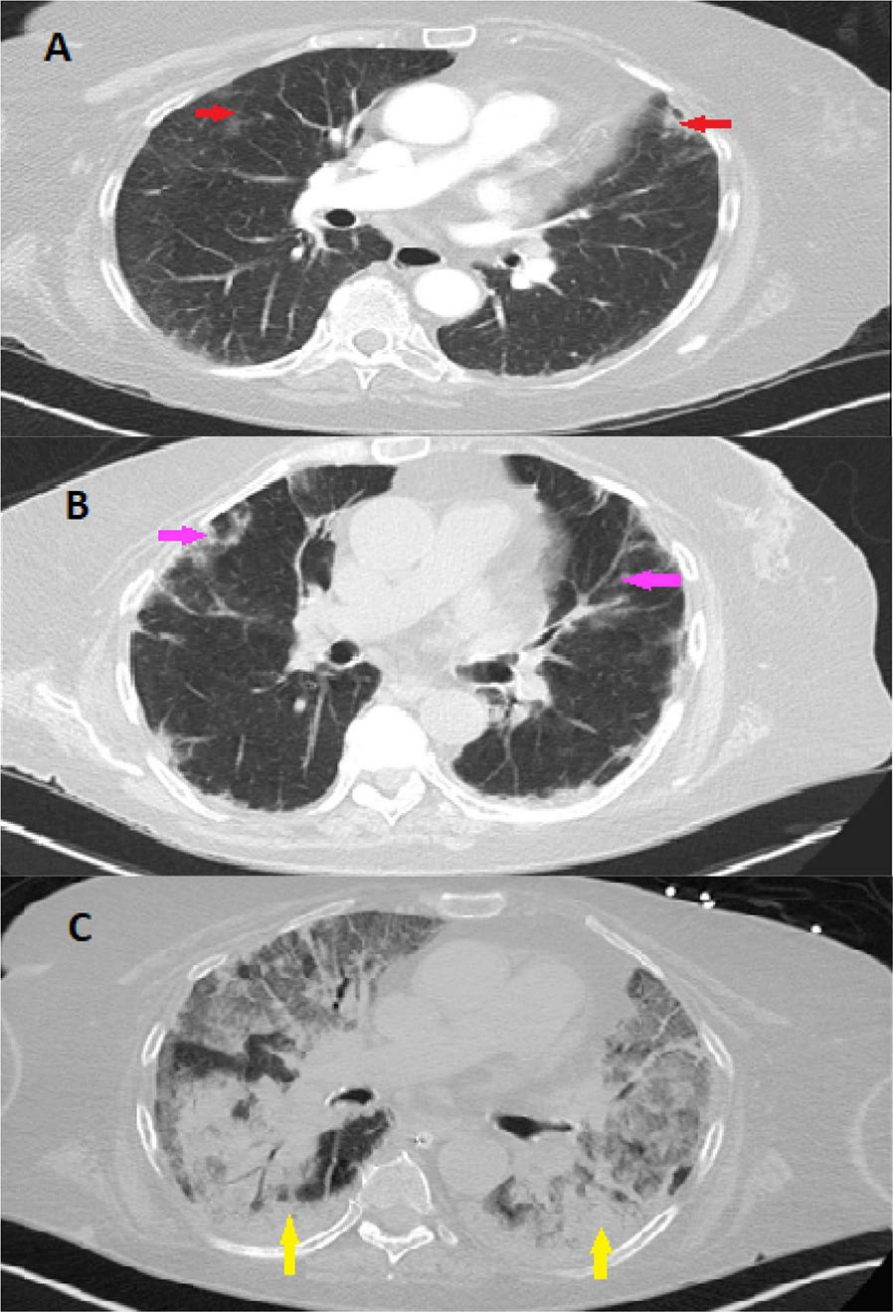
Computed Tomography of the Chest. CT scan of the chest with contrast obtained during the first admission demonstrates mild bilateral patchy infiltrates showing by red arrows (**a**). CT of chest obtained during second admission demonstrates bilateral patchy ground glass densities as showing by pink arrows (**b**). CT of chest during the patients second hospital admission demonstrates worsening consolidative processes within the bilateral lower lobes, more prominently in a peripheral distribution (yellow arrow, **c**)

Given the classic skin manifestations, proximal muscle weakness, and notably elevated CK, a tentative diagnosis of dermatomyositis was made. Immunological screening was positive for anti-nuclear antibody (ANA), melanoma differentiation-associated protein 5 (MDA5), SSA-52 (Ro), and a low C3 complement (Table 2). Diagnostic imaging included an MRI of the right femur that demonstrated multiple scattered areas of proximal muscle edema (Fig. 1), which while nonspecific, was felt to be consistent with an inflammatory myositis. Skin biopsy of the anterior chest was subsequently performed which demonstrated vacuolar interface dermatitis with an increase in dermal mucin (Fig. 2). Computed Tomography (CT) of the chest with contrast demonstrated mild bilateral patchy infiltrates (Fig. 3a). It was not clear as to whether the pulmonary findings were secondary to an infectious etiology such as COVID-19, alternate virus, a bacterium, or other etiologies such as Interstitial Lung Disease or dermatomyositis. For further evaluation an infectious work-up including blood cultures, sputum culture, legionella urinary antigen, and interferon-gamma release assay were performed and all studies were negative. Echocardiogram failed to demonstrate any evidence of vegetations, systolic or diastolic dysfunction, or pulmonary hypertension. She was initiated on oral prednisone 60 mg daily and demonstrated symptomatic improvement in her breathing within 10 days and was tapered to 40 mg after 14 days. She was discharged to a rehabilitation center with plans to continue steroid therapy with adjunctive trimethoprim-sulfamethoxazole for pneumocystis pneumonia prophylaxis. The labs at the time of discharge are mentioned in Table 2.

Unfortunately, two weeks after discharge she presented to our facility in the context of recurrent fever and progressive shortness of breath. She was febrile (38.3 °C), tachypneic (22 breaths per min), with mild hypotension (94/55 mmHg), but an otherwise preserved heart rate (79 beats per minute) and oxygen saturation of 96% on room air. Physical examination was notable only for a persistent, albeit improved V and shawl signs, stable generalized weakness, stable Gottron’s rash and a resolved heliotrope rash. Laboratory diagnostics demonstrated leukopenia with lymphopenia and anemia. Metabolic panel demonstrated a marked acute kidney injury, worsening transaminitis, and an elevated CK and LDH. Given concern for MAS, additional workup was done which revealed marked ferritin and triglyceride elevation. Interleukin 2 receptor (CD25) was within normal limits (Table 2). Diagnostic imaging included a Computed Tomography (CT) of the chest, abdomen and pelvis that demonstrated bilateral patchy ground glass densities and diffuse hepatic steatosis (Fig. 3b). No splenomegaly was noted on the CT scan.

She was initiated on broad spectrum antibiotics given her fever and immunosuppressed status. Antibiotics were subsequently discontinued due to negative blood cultures. For further evaluation of infectious and non-infectious etiologies, a bronchoalveolar lavage (BAL) was performed and demonstrated no evidence of alveolar hemorrhage, negative antigens/ cultures for cytomegalovirus, adenovirus, galactomannan, legionella, acid-fast bacillus, or histoplasmosis. Cell count and gram stain were equally unremarkable. Intriguingly, however, (1,3)-BD-glucan was noted to be positive and cultures demonstrated positivity for aspergillum, for which she was initiated on Voriconazole. Given her worsening clinical status despite antibiotic therapy and concern for MAS, she was initiated on high-dose methylprednisolone (1 g daily) for three days followed by 80 mg IV daily. She was also initiated on intravenous immunoglobulin 400 mg/kg/ day for a five-day course. Despite such therapy the patient developed worsening hypoxemic respiratory failure necessitating mechanical ventilation. Bone marrow biopsy was deferred. Repeat imaging (CT) demonstrated a new, marked consolidative processes within the bilateral lower lobes in a peripheral distribution with pleural sparing (Fig. 3c). Given the patients deteriorating clinical condition with adjunctive worsening of the inflammatory markers and worsening CT findings despite treatment of aspergillus, she underwent repeat bronchoscopy that demonstrated Pseudomonas for which she was initiated on Piperacillin-Tazobactam therapy. Unfortunately, her hospital course was complicated by worsening renal function necessitating continuous renal replacement therapy, worsening cytopenia with marked anemia, atrial fibrillation with rapid ventricular response, and hypotension requiring pressor support. Her respiratory status demonstrated continued decline despite ventilator support and she ultimately passed in the setting of pulseless electrical activity arrest due to hypoxemic respiratory failure.

## Discussion and conclusions

Our case describes a newly diagnosed MDA5-positive dermatomyositis complicated by MAS with recent diagnosis of COVID-19 infection. Several studies reported the association of MAS in rheumatological disease [3], while a limited number of reports have described the association of MDA5-positive dermatomyositis and MAS. Nevertheless, the overall incidence of MAS associated by dermatomyositis remains rare [3, 10–12].

COVID-19 infection may present with a multitude of pulmonary findings including multifocal, diffuse ground glass opacities. Similarly, the pulmonary manifestations of dermatomyositis may present with patchy ground glass opacities, similar to commonly seen findings in COVID-19 infection, but also present with basilar consolidation, septal thickening, honeycombing, and irregular linear opacities. Our patient had negative infectious work-up on her first admission, and given the symptomatic improvement by prednisone, the pulmonary pathology was thought to be secondary to newly diagnosed MDA5 positive dermatomyositis. On the patients second presentation, the constellation of fevers and bilateral patchy ground-glass opacities was felt to be secondary to progressive Interstitial Lung Disease in the context of underlying MDA5 positive dermatomyositis, accompanied by an underlying cytokine storm. While the patient was indeed immunocompromised and Pneumocystis pneumonia (PCP) was considered in the differential, the imaging findings appeared atypical from classic PCP pneumonia and the patient was noted to be previously discharged on suppressive doses of Bactrim for prophylaxis. BAL failed to demonstrated evidence of alveolar hemorrhage and indeed was negative for cytomegalovirus, adenovirus, galactomannan, legionella, acid-fast bacillus, or histoplasma, with an unremarkable cell count and gram stain. Although she was positive for (1,3)-BD-glucan and cultures demonstrated positivity for aspergillus, the patients clinical condition deteriorated despite Voriconazole therapy. She also developed worsening inflammatory markers and CT findings. Repeat BAL demonstrated pseudomonas. Despite antibiotic treatment she demonstrated ongoing clinical deterioration with further laboratory abnormalities (hyperferritinemia, cytopenia) raising concern for MAS. Bone marrow biopsy was planned but ultimately was deferred in the setting of worsening hyperemic respiratory failure. At this point patient developed worsening renal function required continuous veno-venous hemofiltration (CVVH).

The diagnosis of MAS remains a clinical challenge. Although multiple diagnostic criteria for HLH/MAS have been developed—HLH-2004, SLE-MAS, and the novel 2016 criteria for MAS complicating systemic juvenile idiopathic arthritis (sJIA)—a uniform diagnostic criteria for MAS remains elusive [5], with no diagnostic criteria identified for MAS associated with MDA5-positive dermatomyositis. The most commonly used diagnostic criteria for HLH (i.e., “HLH-2004”) include fever, splenomegaly, cytopenia in at least two cell lines, hypertriglyceridemia and/or hypofibrinogenemia, tissue presentation of hemophagocytosis, minimal or no natural killer cell activity, serum ferritin concentration > 500 ng/mL and elevated levels of soluble CD25 (> 2 standard deviations above the normal mean; typically > 2400 IU/mL) [13]. Hemophagocytosis leads to the presence of red blood cells, white blood cells or platelets within the macrophage cytoplasm which can be seen on lymph node, spleen, liver, or bone marrow biopsy [14]. Lung opacities secondary to inflammatory processes can be seen in MAS. Our patient met four criteria for the diagnosis of HLH including fever (Temperature 40 °C), bicytopenia with hemoglobin 5.1; platelet 83,000, elevated triglyceride > 1100, and elevated ferritin > 16,500 (Table 3). All criteria may not be met on the initial presentation due to lack of some features (e.g. hemophagocytosis) early in the disease process and it is unlikely that every case of HLH will meet diagnostic criteria. Given the high mortality of HLH, and the need to “capture” false negative cases, a modified diagnostic criteria (Table 4) created and reported to be used for diagnosis of HLH and support the need for initiation of HLH therapy. Modified diagnostic criteria include at least three of four clinical findings of fever, splenomegaly, cytopenia, and hepatitis plus at least one of four abnormalities, including hemophagocytosis, increased ferritin, elevated soluble IL2Ra (sCD25), and absent or very decreased NK cell function. Hypertriglyceridemia and hypofinbrinogenemia can also support the diagnosis [15]. Our patient had fever, cytopenia, and hepatitis plus elevated ferritin and eventually developed renal failure required dialysis and respiratory failure required intubation which meets the modified criteria. Unfortunately, a bone marrow biopsy could not be performed due to the patient's declining respiratory status.

**Table 3:** Comparison of our patient and HLH-2004 criteria

HLH-2004 criteria	Our patient
Fever ≥ 38.5 °C	Fever with temperature at 40 °C
Peripheral blood cytopenia, with at least two of the following: hemoglobin < 9 g/dL (for infants < 4 weeks, hemoglobin < 10 g/dL); platelets < 100,000/microL; absolute neutrophil count < 1000/microL	Hemoglobin 7.8 gm/dl and platelet count 83 k/µL
Hypertriglyceridemia (fasting triglycerides > 265 mg/dL) and hypofibrinogenemia (fibrinogen < 150 mg/dL)	Triglyceride level 1100 mg/dl
Ferritin > 500 ng/mL	Ferritin > 16,500 ng/mL

**Table 4:** Modified diagnostic criteria

1	Molecular diagnosis of hemophagocytic lymphohistiocytosis (HLH) or X-linked lymphoproliferative syndrome (XLP)
2	Or at least 3 of 4 of the following Fever Splenomegaly Cytopenias (minimum 2 cell lines reduced) Hepatitis
3	And at least 1 of 4 of the following Hemophagocytosis ↑ Ferritin ↑ sIL2Rα (age based) Absent or very decreased NK function
4	Other results supportive of HLH diagnosis Hypertriglyceridemia Hypofibrinogenemia Hyponatremia

Cytokine storm appears to be critical to the pathophysiology of MAS. Overexpression of inflammatory cytokines leads to the activation of macrophage, natural killer (NK), and cytotoxic T lymphocytes with resultant multi-organ dysfunction [14]. In the setting of infection or inflammatory state, NK cells and cytolytic T lymphocytes may lyse infected and activated antigen presenting cells. Defects in the cytolytic function of NK cells, a presumed feature in many cases of MAS, may induce a pro-inflammatory cytokine cascade and result in immune system hyperstimulation leading to multiorgan failure seen in MAS [16]. Immune suppression is a central feature of MAS management. Glucocorticoids are the mainstay of treatment in MAS but in select cases remain ineffective in controlling the cytokine storm. Additional treatment including immunosuppression, IV immunoglobulin, plasma exchange, etoposide and biologic agents have been used in reported cases of dermatomyositis complicated by MAS [10]. Anti-interleukin (IL)-1, IL-6, and tumor necrosis factor (TNF) therapies have shown improved outcomes in MAS [17] with resultant improvement in ferritin, and triglyceride serum levels [18]. Our patient required intubation eventually leading to death despite steroid and IV immunoglobulin therapy.

To date there are no noted cases pertaining to MDA5-positive dermatomyositis or MDA5-positive dermatomyositis complicated by MAS with recent diagnosis of COVID-19. The patient presented in this case was diagnosed with COVID-19 two months prior to presentation at which time she was managed supportively. She subsequently developed progressive weakness, increased fatigue, and shortness of breath leading to hospital admission, and subsequent diagnosis of MDA5-positive dermatomyositis. Tanboon and colleagues presented a case of dermatomyositis found to have positive anti-small ubiquitin-like modifier-1-activating enzyme (anti SAE) antibody in the context of COVID-19 [19]. They hypothesized that COVID-19 mediated Type I interferon activation may precipitate interferon mediated myofiber damage [19]. Intriguingly, three immunologic epitopes with high sequence identity to SARS-COV-2, the causative virion of COVID-19, were identified in patients with autoimmune dermatomyositis, suggesting an overlapping pathophysiological mechanism between severe COVID-19 infection and dermatomyositis [20]. Anti- IL-1 and IL-6 treatment which has been shown promising outcomes in MAS treatment, also demonstrated significant improvement in patient’s symptoms infected with COVID-19 [15, 21]. At this time the potential contribution of COVID-19 infection to the development of MDA5-positive dermatomyositis or MDA5-positive dermatomyositis complicated by MAS remains unknown. The hyperinflammatory state in COVID-19 infection may play a role in development of MAS associated with dermatomyositis. These findings highlight that COVID-19 infection may contribute to the development of dermatomyositis through a predisposing inflammatory state.

Anti-MDA5 dermatomyositis may be associated with the development of MAS. Early diagnosis and treatment of MAS especially in the context of anti-MDA5 antibody positive dermatomyositis is essential. Further investigation is required to correlate the association between anti-MDA5 antibody and development of MAS. COVID-19 infection may play a role in development of dermatomyositis or MAS, however the potential contribution of COVID-19 infection to the development of either disease remains unknown and further investigations are needed to elucidate the association.

## Data Availability

All clinical data used for writing this case report are included in this published article (tables, figures).

## Abbreviations

AST: Aspartate aminotransferase
ALT: Alanine aminotransferase
ALP: Alkaline phosphatase
ANA: Anti-nuclear antibody
COVID-19: Coronavirus disease 2019
CK: Creatine kinase
CRP: C-reactive protein
CT: Computed tomography
CVVH: Continuous veno-venous hemofiltration
ESR: Erythrocyte sedimentation rate
HLH: Hemophagocytic lymphohistiocytosis
IL: Interleukin
IV: Intra venous
SJIA: Juvenile idiopathic arthritis
LDH: Lactate dehydrogenase
MAS: Macrophage activation syndrome
MDA5: Melanoma differentiation-associated protein 5
NK: Natural killer
PCP: Pneumocystis pneumonia
SARS-COV-2: Severe acute respiratory syndrome coronavirus 2
SLE: Systemic lupus erythematous
TNF: Tumor necrosis factor
